# Dielectric Spectroscopy Analysis of Liquid Crystals Recovered from End-of-Life Liquid Crystal Displays

**DOI:** 10.3390/molecules26102873

**Published:** 2021-05-12

**Authors:** Ana Barrera, Corinne Binet, Frédéric Dubois, Pierre-Alexandre Hébert, Philippe Supiot, Corinne Foissac, Ulrich Maschke

**Affiliations:** 1 CNRS, INRAE, Centrale Lille, UMR 8207—UMET—Unité Matériaux et Transformations, Université de Lille, F-59000 Lille, France; ana-luisa.barrera-almeida@univ-lille.fr (A.B.); corinne.binet@univ-lille.fr (C.B.); philippe.supiot@univ-lille.fr (P.S.); corinne.foissac@univ-lille.fr (C.F.); 2 UR 4476, UDSMM, Unité de Dynamique et Structure des Matériaux Moléculaires, Université du Littoral Côte d’Opale, 59 379 Dunkerque, France; frederic.dubois@univ-littoral.fr; 3 UR 4491, LISIC, Laboratoire d’Informatique Signal et Image de la Côte d’Opale, Université du Littoral Côte d’Opale, 59 379 Dunkerque, France; pierre-alexandre.hebert@univ-littoral.fr

**Keywords:** nematic liquid crystals, recycling, dielectric properties, diamond nanoparticles, Jonscher

## Abstract

In the present work, the dielectric properties of recycled liquid crystals (LCs) (non-purified, purified, and doped with diamond nanoparticles at 0.05, 0.1, and 0.2 wt%) were investigated. The studied LC mixtures were obtained from industrial recycling of end-of-life LC displays presenting mainly nematic phases. Dielectric measurements were carried out at room temperature on a frequency range from 0.1 to 10^6^ Hz using an impedance analyzer. The amplitude of the oscillating voltage was fixed at 1 V using cells with homogeneous and homeotropic alignments. Results show that the dielectric anisotropy of all purified samples presents positive values and decreases after the addition of diamond nanoparticles to the LC mixtures. DC conductivity values were obtained by applying the universal law of dielectric response proposed by Jonscher. In addition, conductivity of the doped LC mixtures is lower than that of the undoped and non-purified LC.

## 1. Introduction

At present, liquid crystals (LCs) represent a very important value in display systems. LCs exhibit an organized structure between solid and isotropic liquid states. These materials behave physically like a liquid, but at the same time, exhibit the properties of an organized medium. In general, depending on position, chirality, and order of orientation of the LC molecules, three major mesophases are found: nematic, smectic, and cholesteric [1,2]. Mesogens can have various structures; they can be calamitic, discotic, banana shaped, or LC polymers. The recycled LCs studied in this report mainly present a nematic phase and their molecules exhibit an elongated shape, represented by rods distributed in an ordered way. In this phase, the long axes of the molecules are arranged approximately in the same direction, but their positions are freely distributed. This means that they have only one order of orientation, and no privileged position in space. This preferential direction is referred to as the director of the nematic LC, often represented by a vector noted $\vec{n}$ [3].

The orientational order is one of the most important features of LCs, providing an anisotropic character, i.e., LCs give different responses depending on the direction in which the external field (electric, magnetic) is applied [1,2]. Thus, in a nematic LC, ε_//_ represents the dielectric permittivity when the electric field is parallel to $\vec{n}$, and ε_⊥_ corresponds to the case when the electric field is perpendicular to $\vec{n}$ [4]. Magnitude and sign of the dielectric anisotropy (Δε) are two of the most influential factors in the selection of nematic LC for a particular application [5].

The remarkable point of the approach presented here, unique in Europe, is the attempt to revalorize LCs present in end-of-life LCDs. Nowadays, LCs represent an important economic value of the recycling system of LCDs. The reuse of these organic molecules could become a profitable basis since it permits to preserve the value of these materials. On a global level, the only pilot plant known for “total waste-free processing” of end-of-life LCDs is located in Taiwan, established by researchers of the Industrial Technology Research Institute (ITRI). After more than a decade of research, they developed a recycling process to obtain LCs with less than 1 ppb of impurities [6]. At this purity level, LCs can be easily reused to manufacture new LCDs or smart windows.

The presence of ionic impurities in LC mixtures causes a large number of undesirable effects; for example, they tend to increase the electrical conductivity, which can lead to alterations in optical and electro-optical properties (sticking and flickering images, slow response in electro-optical devices) [7]. Therefore, it is essential that the recovered LC mixtures possess high purity so that the recycled LCs could regain new life.

To achieve purification of LCs, several physicochemical methods exist which are often expensive, difficult, and/or time consuming [8]. Once purified, even using a sophisticated purification method that would result in highly purified LCs, ionic contamination can still occur, for example, during the manufacture of new LC-based devices [7,8,9]. Based on this observation, establishment of a persistent purification method is mandatory.

During the last 20 years, one of the most promising and studied ways to capture ionic impurities consists to add inorganic or organic nanomaterials as ion-adsorbent materials to LCs. In general, adding a small amount of nanomaterials (concentrations lower than 1 wt%) will be sufficient to significantly improve electro-optical, magnetic, and dielectric properties of LCs [10]. The most commonly investigated dopants were noble metals [11], ferroelectrics [12], semiconductors, magnetics or insulators nanoparticles [13,14,15], carbon-based nanomaterials (fullerenes, graphenes, carbon nanotubes [16]), carbon dots [8,9], and diamond nanoparticles (DNPs) [17,18,19,20]. After addition of such nanoparticles to LCs, two main effects have been reported: either the electrical conductivity increases or decreases.

In this report, dielectric properties (ε’, ε”, Δε) of non-purified, purified, and nanoparticle-doped recycled LCs mixtures are investigated. A particular interest is given to DNPs to study their impact on mobile ions present in the recovered LCs. DNPs belong to one of three allotropic forms of carbon [21]. They are interesting materials for their mechanical, thermal, and optical characteristics. Moreover, they are chemically stable and non-conductive, non-toxic and possess high surface areas, inert surface, and tunable surface structures [22].

## 2. Results and Discussion

The complex dielectric permittivity (ε*) is given by Equation (1) [4]:

ε*(ω) = ε’(ω) *−i*ε”(ω), (1)

where ω stands for the angular frequency (ω = 2πf), f is the frequency of the measuring electric field, *i* = $\sqrt{-1}$, and ε’ and ε” represent the real and imaginary parts of ε*, respectively. The dielectric anisotropy is obtained by Equation (2):

Δε = ε’_//_ − ε’_⊥_. (2)

### 2.1. Dielectric Measurements: Effect of the Amplitude of the Oscillating Voltage

**Figure 1:**
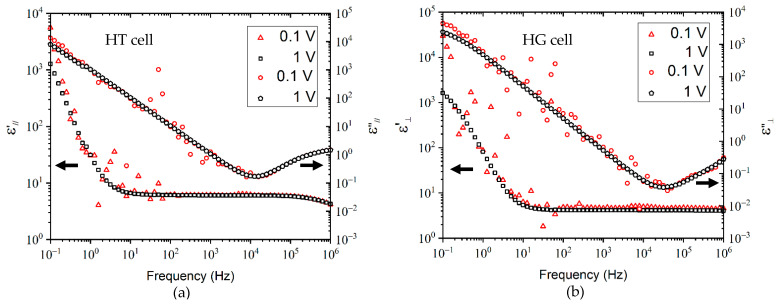
Dielectric spectra of a non-purified LC mixture at room temperature (20 °C), using 20 µm cells in (**a**) homeotropic and (**b**) homogeneous alignment, for two amplitudes of the oscillating voltage: 0.1 and 1 V.

The quality of the linear dielectric response depends on the studied material, on the device characterization, and also on the amplitude of the sinusoidal voltage to be applied (Ve). Preliminary measurements were carried out at room temperature for V_e_ = 0.1 and 1 V. Figure 1 presents the spectra ε’ and ε” as a function of frequency of a non-purified LC mixture in homogeneous (HG cell) and homeotropic (HT cell) alignments.

The spectra observed for V_e_ = 0.1 V show a significant sensitivity of the dielectric signal to external electromagnetic disturbances. When V_e_ = 1 V, this phenomenon appears much weaker, although the permittivities are quite close to those obtained for V_e_ = 0.1 V. The difference between the two sets of measurements can be quantitatively assessed by the parameter δ (distortion measure), given by Equation (3):

(3) $$\delta \left(\varepsilon \right)=\frac{100}{m}\cdot \frac{\sum^{\;}\left|\log\left(\varepsilon_{1V}\left(f\right)\right)-\log\left({\;\varepsilon }_{0.1V}\left(f\right)\right)\right|}{\max\left(\log\left(\varepsilon_{1V}\left(f\right)\right)\right)-\min\left(\log\left(\varepsilon_{1V}\left(f\right)\right)\right)}\;\;,$$

where m stands for the number of measurement frequencies. δ represents a mean absolute difference, expressed as a percentage of the maximum range of the 1V spectra. The δ data were found between 3% and 8.5% (δ(ε’_⊥_) = 8.5%; δ(ε”_⊥_) = 4.6%; δ(ε’_//_) = 3.2%; and δ(ε”_//_) = 3.4%). Consequently, a value of 1 V for the sinusoidal voltage of the electric field measurement has been chosen for all measurements presented in this work.

### 2.2. Effect of Alignment on Dielectric Properties of Non-Purified LCs Mixtures

**Figure 2:**
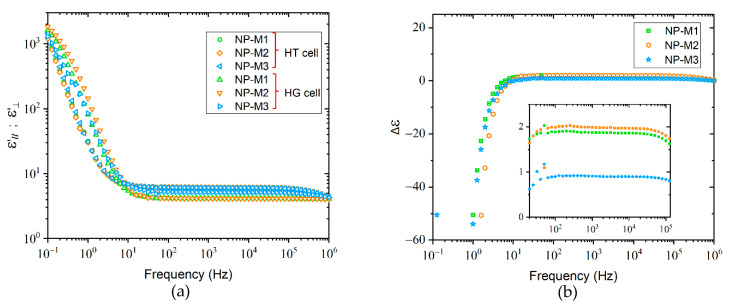
(**a**) Relative permittivity and (**b**) dielectric anisotropy of three non-purified LC mixtures as function of frequency. Measurements were taken at 1 V and room temperature (20 °C) with 20 µm cells in homogeneous and homeotropic alignments. NP-M stands for non-purified LCs mixtures.

Figure 2 shows the real part of the complex permittivity in homogeneous and homeotropic alignments of three non-purified LC mixtures (NP-M1, NP-M2, and NP-M3). A fundamental point for the reuse of a recycled product is the reproducibility of its properties. The characteristics of the different mixtures must be almost identical to consider a future reuse. It is therefore necessary to measure their characteristics and mainly their permittivities.

In Figure 2a, two distinct frequency regions can be appreciated:

The first one is situated between 0.1 and 10 Hz. When frequency decreases, a significant increase of the values of ε’_//_ and ε’_⊥_ is observed, to reach values higher than 10^3^. This phenomenon can be attributed to the space charge polarization that is normally created at the interfaces formed between the electrodes of the cell and the LCs known as electrode polarization, and to a contribution of the electrical conductivity produced by the ionic impurities present especially in the non-purified (NP) samples. Interestingly, in this frequency range, all ε’_//_ as well as ε’_⊥_ curves of NP-M1-3 are superimposed, exhibiting an overall shift between ε’_//_ and ε’_⊥_. All NP-LCs mixtures possess a negative dielectric anisotropy because the curve of ε’_⊥_ has larger values than ε’_//_ as illustrated in Figure 2b.

The second one is situated between 10 and 10^6^ Hz. For each LC mixture, permittivity is independent of frequency. At such frequencies, the ionic impurities cannot follow the periodic inversion of the electric field. Contrary to the phenomenon observed between 0.1 and 10 Hz, the dielectric anisotropy remains positive (ε’_//_ > ε’_⊥_). The inset of Figure 2b presents an extended view of Δε in the frequency range between 30 and 10^5^ Hz in order to visualize the different dielectric anisotropy values. There are found as: 1.87, 1.99, and 0.90 for NP-M1; NP-M2; and NP-M3, respectively. 

It was expected to obtain identical phenomena in both frequency domains. However, a clear difference of the results between these two frequency ranges and especially a sign inversion of Δε is observed for the three LC mixtures.

In terms of optical appearance, the extracted LCs present a black coloration that is not typical for nematic LCs (see Figure 6c in Section 3.2). It is obvious that during the industrial recovering procedure applied to extract the LCs, several sources of contamination appeared. For example, the organic solvent used can capture unwanted molecules by solubilizing a significant number of organic and inorganic impurities. In addition, impurities such as dust, water, adhesive residues, scraps, and other materials, present in a storage and treatment plant of Waste Electrical and Electronic Equipment (WEEE), could be added to the solution containing the extracted LCs.

For all reasons mentioned above, purification of these mixtures is a mandatory step for the possible reuse of the LCs.

### 2.3. Dielectric Anisotropy of Purified LC Mixtures and Doped with DNPs

**Figure 3:**
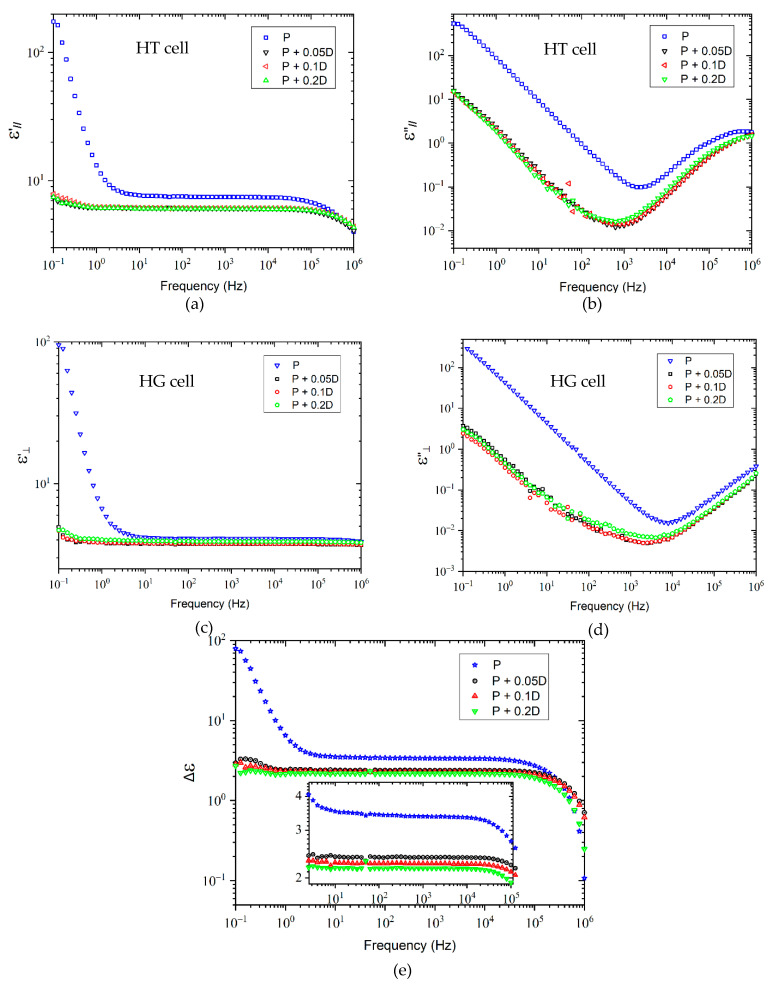
Dielectric permittivity of purified LCs doped with different concentrations of DNPs (0.05, 0.1, and 0.2 wt%): (**a**) real and (**b**) imaginary parts in homeotropic alignment, (**c**) real and (**d**) imaginary parts in homogeneous alignment, and (**e**) dielectric anisotropy. The spectra were measured under identical experimental conditions (P stands for purified LC mixtures; P + 0.05D, P + 0.1D, and P + 0.2D correspond to purified LC mixtures doped with 0.05, 0.1, and 0.2 wt% of DNPs, respectively).

After purification, all LC mixtures exhibit quite comparable properties, the same optical appearance and equivalent dielectric anisotropy values at 1 kHz (see Figure S1 in Supplementary Materials). Therefore, the results of doping for only one of these LC mixtures will be presented in the following sections in order to avoid redundancy. The dielectric properties of such a representative purified LC mixture doped with three different concentrations of DNPs (0.05, 0.1, and 0.2 wt%) were measured. Figure 3 illustrates the real (dielectric constant ε’) and imaginary (dielectric loss ε”) parts of the complex dielectric permittivity, and the dielectric anisotropy.

Figure 3a,c show two different regions of the real part of the dielectric permittivity for homeotropic and homogeneous alignments. At low frequencies between 0.1 and 10 Hz, when frequency decreases, a significant increase of ε’ is observed for the purified LC mixture. As explained earlier, this phenomenon is a combination of electrode polarization and electrical conductivity produced from ionic impurities still present in the purified LC sample. On the other hand, doped samples do not reveal a significant increase of ε’ with decreasing frequency. For samples doped with DNPs, a strong flattening of the slopes of the ε’ curves is observed compared to the purified LC sample. At frequencies between 10 and 10^5^ Hz, ε’ remains almost constant for all samples since at such frequencies, the ions as impurities can no longer follow the periodic inversion of the electric field [23]. It should be noted that above 10^5^ Hz, ε’decreases for all samples with increasing frequency, for the homeotropic alignment.

Figure 3b,d also present two different regions of the imaginary part of the dielectric permittivity: at high frequencies, a relaxation mechanism appears in the imaginary part of ε*. Nevertheless, this relaxation phenomenon seems not to be associated to any LCs molecules. It might correspond to the relaxation of the measuring cell due to the resistance of the ITO layer [24], in relationship with parasitic impedances caused by connectors, cables, etc., which become important at frequencies above 100 kHz [4]. The dielectric response of the analyzed LCs is hidden by the response of the measuring cells. This effect has already been discussed by other authors [25,26,27,28].

The addition of DNPs to LC mixtures decreases ε’and ε’’ by more than two orders of magnitude at low frequencies, compared to the purified sample.

A comparison between Δε data from Figure 2b with the corresponding result for the purified LC mixture in Figure 3e reveals an increase of the dielectric anisotropy of the latter, due to the important reduction of the amount of impurities. In the case of the NP samples, the orientational effects are perturbed due to the presence of impurities.

According to the data presented in Figure 3e, the dielectric anisotropy of the purified sample decreases by roughly 30% by adding DNPs. Δε presents positive values for all samples in the whole frequency range, since the longitudinal component of the dielectric constant is always greater than the perpendicular one (Figure 3a,c). A small dependence between the amount of DNPs present in the LC mixtures and the decrease of the dielectric anisotropy is also observed, such that the larger the amount of nanoparticles, the lower the dielectric anisotropy. However, the y-axis scale of this figure does not allow to appreciate this behavior. Therefore, an inset applying a different scaling has been added to better illustrate the evolution of Δε. More precisely, the following Δε data (at 1 kHz) are found: 3.36; 2.38; 2.25; and 2.17 for P; P + 0.05D; P + 0.1D; and P + 0.2D wt% LCs mixtures, respectively. These values are within the range of dielectric anisotropy values of LCs mixtures developed by Merck [29,30]. The decrease of Δε may be related to the dipole–dipole interactions between the nematic LC molecules and the DNPs, and has already been reported by other authors [19,20]. According to the literature, DNPs not only possess a distinct value of the polarizability α (α = 1.95 × 10^−40^ C^2^⋅m^2^⋅J^−1^) [31] but also a small permanent dipole moment, compared to that of LC molecules. By taking into account the volumetric density (*ρ* = 3.5 × 10^3^ kg⋅m^−3^) and its polarization (P = 10^−7^ C⋅m^−^^2^), the permanent dipole moment of diamond can be calculated as: µ = PV = 5.71 × 10^−37^ C⋅m (V corresponds to the unit volume). Therefore, ion-dipole interactions are present in LC-DNPs mixtures, together with dipole–dipole interactions between LC molecules.

In summary, the highest value of dielectric anisotropy is obtained by removing all impurities present in LCs mixtures. However, the removal process is a rather complex task due to various sources and natures of the impurities. Therefore, it is more feasible to decrease the amount of impurities by typical purification methods and to decrease the electrical conductivity furthermore by adding DNPs.

### 2.4. Frequency Dependence of Real Conductivity for Non-Purified, Purified and Nanoparticle Doped LC Mixtures

The complex conductivity (σ*) is an alternative representation of the dielectric properties, allowing to better understand or identify the aspects and phenomena of polarization and charge transport (ions, impurities). It is represented by Equation (4):

σ* (ω) = σ’(ω) + *i*σ”(ω), (4)

where σ’ and σ” are the real and imaginary parts of this function. The complex functions ε*(ω) and σ*(ω) are related by Equation (5) [4]:

σ* (ω) = *i*ωε_0_ε*, (5)

where ε_0_ corresponds to the dielectric permittivity of vacuum (8.85418 × 10^−12^ F·m^−1^). Real and imaginary parts of the complex conductivity are given by Equations (6) and (7):

σ’(ω) = ωε_0_ε”(ω), (6)

σ”(ω) = ωε_0_ε’(ω). (7)

In this report, the conductivity spectra were analyzed applying Jonscher’s universal power law [32]. This model, known as the Universal Dielectric Response (UDR), is widely used to analyze the frequency dependence of the real part of the complex conductivity. The equation of Jonscher can be expressed as:

(8) $$\sigma ’=\sigma_{\mathrm{DC}}+A\omega^{n}.$$

Equation (8) can also be written in the following form, considering:$\;A\omega^{n}=$σ_DC_${\left(\frac{f}{f_{c}}\right)}^{n}$

(9) $$\sigma ’=\sigma_{\mathrm{DC}}\left(1+{\left(\frac{f}{f_{c}}\right)}^{n}\right),$$

where σ_DC_ represents the DC conductivity, *f*_c_ stands for the characteristic frequency, and n is a parameter, which represents the degree of interaction between the mobile ions and their surroundings. The value of n is normally situated between 0 and 1, however some authors have found values where n > 1 [33,34,35,36]. Many other models are available to analyze the frequency dependence of the complex conductivity like Cole–Cole, Cole–Davidson, Havriliak–Negami, or Kohlrausch–Williams–Watts functions transformed beforehand into their conductivity representations [37,38,39,40,41]. Dyre’s approach based on a random free energy barrier model can also be considered. The latter model is often applied for disordered ion conducting solids and ionic liquids [42,43].

**Figure 4:**
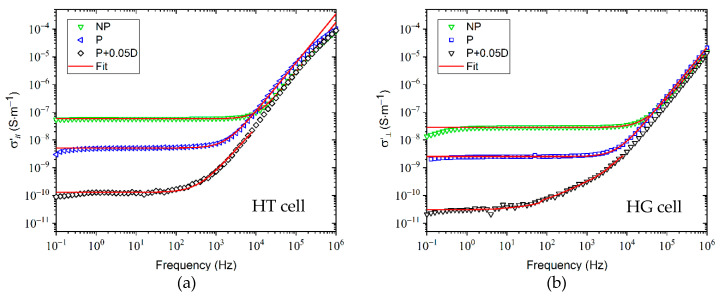
Real part of the complex conductivity in (**a**) homeotropic and (**b**) homogeneous alignments as a function of frequency of non-purified, purified, and DNP-doped (0.05 wt%) LC mixtures. The experimental data are represented by symbols and the red lines show the curves obtained applying Jonscher’s model. NP corresponds to non-purified; P is for purified; and P + 0.05D stands for LC mixtures doped with 0.05 wt% of DNPs.

Figure 4 illustrates the real component (σ’) of the complex conductivity (σ*) in homogeneous and homeotropic alignments on a frequency range from 0.1 Hz to 1 MHz, obtained from Equation (6). The dielectric conductivity represents a process that combines jumps, mobility, and transport of charge carriers present in the material [4,36].

The σ’ curves of NP- and P-LC mixtures in homogeneous and homeotropic alignments show two main regions: (1) a plateau region in the frequency range from 1 Hz to 1 kHz, which is almost independent of frequency. Hence, the DC conductivity can be estimated by the Jonscher’s power law; (2) at frequencies above 1 kHz, the conductivity is frequency dependent. According to Jonscher, this dependence may be due to relaxation phenomena originating from mobile charge carriers [44].

At lower frequencies (<1 Hz), σ’ starts to decrease. This phenomenon is attributed to electrode polarization. NP-LC mixtures exhibit the highest conductivity compared to the other samples. The fitting curves presented by red lines agree well to the experimental values for all samples. It is important to note that the conductivity values of the doped samples are extremely low (in the range of 10^−10^ and 10^−11^ S·m^−1^), and almost at the level of sensitivity of the measuring device, explaining the noisy character of the spectra.

The fitting results have been listed in Table 1.

In the homeotropic alignment, n presents values greater than 1 (n > 1) for all samples. In the homogeneous alignment, n is greater than 1 as well for both P- and NP-LC mixtures. For the doped mixtures, the values of n are smaller than 1 and are therefore situated within the limits proposed by Jonscher.

The obtained values for n depend on sample nature and temperature. In our case, although the temperature was identical for all experiments, the viscosity of the LC mixtures varied with the addition of DNPs. When n becomes smaller than 1, the process of charge carrier displacement involves a translational motion with a sudden jump. When n > 1, the movement involves a localized jump of the charge carriers that will leave their neighborhood completely [36]. Some research groups reported about n values with n > 1. Models were developed for this case like that of Kılıç et al. [35], designated as Super Linear Power Law (SLPL), and the Quantum Mechanical Tunneling (QMT) model for the case 0.7 ≤ n ≤ 1.

**Figure 5:**
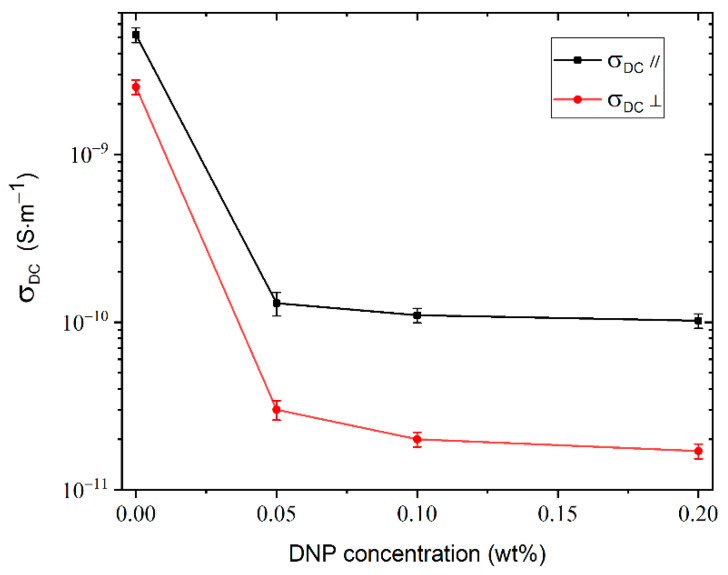
Variation of DC conductivity as a function of concentration of DNPs added to a purified LC mixture.

The variation of the DC conductivity as a function of the concentration of DNPs is presented in Figure 5. The DC conductivity of purified LCs decreases significantly with the addition of DNPs for both alignments. There is a decrease of about 98% between the purified sample and the one doped with 0.05% DNPs. For the three concentrations of nanoparticles, the decreases are small, and the σ_DC_ values are close together. However, this means that the number of mobile ions decreases when the concentration of nanoparticles increases. The origin of this decrease is due to the adsorption of anions and cations on the surface of the spherical DNPs.

The range of electrical conductivity of thermotropic LCs is comprised between 10^−7^–10^−13^ S·m^−1^, depending on the LC material. The values of electrical conductivity for the well-known LCs 5CB and E7 are generally in the range from 10^−7^ to 10^−8^ S·m^−1^ [8]. Purified LCs from our work present a σ_DC //_ of 5.1 × 10^−9^ and a σ_DC__⊥_ of 2.5 × 10^−9^ S⋅m^−1^. These values are therefore situated within the range of conventional nematic LCs mixtures.

## 3. Materials and Methods

### 3.1. Materials

**Figure 6:**
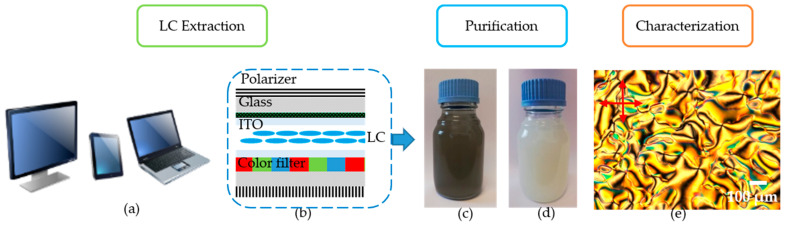
(**a**) End-of-life LCD, (**b**) LCD display composition, (**c**) Non-purified and (**d**) purified LCs mixtures, and (**e**) Texture of purified LC mixtures observed under polarizing optical microscope (POM) Olympus BX60 (Olympus Corporation, Tokyo, Japan), presenting a nematic Schlieren texture. Conditions: LC sample sandwiched between un-aligned glass and coverslip; crossed polarizers; and room temperature.

The recycled LCs mixtures were supplied by the French recycling company ENVIE^2^E, Lesquin, France. An orderly and manual dismantling line of LCD panels is set up for recycling purposes. In order to extract LCs, end-of-life LCD panels (Figure 6a,b) are opened and exposed to a bath of an ultrasonic activated organic solvent. The details of the extraction process are described in a patent [45]. The advantages of this extraction method lie in the recovery speed of LC molecules, and the relatively low contamination effects.

The resulting solution contains LC molecules, the organic solvent, as well as organic and inorganic impurities, especially ions. The solid impurities were filtered out, followed by evaporation of the solvent under primary vacuum (solvent recycling). The samples containing the recovered LCs present an atypical green-black color (shown in Figure 6c). This color might be attributed to the dissolution of the glue used to assemble the two glass plates into the LCD slab and/or other materials dissolved during the extraction process. In order to increase the purity of the LC mixtures, other purification steps are necessary.

Three different LC mixtures were used in this study. Each mixture corresponds to a recovery period of 4 months during one year, thus: Mixture 1 (1–4 months), Mixture 2 (5–8 months), and Mixture 3 (8–12 months). It should be noted that these mixtures were extracted from a large number of end-of-life LCD screens exhibiting highly heterogeneous nature: televisions, computers, and tablets with totally different types, sizes, brands, and years of production.

### 3.2. Purification and Characterization of LC Mixtures

Several distillation and chromatographic steps were used to remove the remaining impurities from the recycled LCs mixtures. The purified LCs present a nematic phase at room temperature (Figure 6c,e) and the clearing temperature is situated around 80 °C.

### 3.3. Addition of DNPs to Purified LC Mixtures

The DNPs used in this study present an average size of diameter lower than 10 nm. They were purchased from Sigma-Aldrich (Saint-Quentin Fallavier, France) and no further purification has been performed. These DNP were added to purified LCs at three different concentrations: 0.05; 0.1; and 0.2 wt%. Before use, to ensure good dispersion, these mixtures were placed in an ultrasonic bath (Elmasonic S30H, Elma Schmidbauer GmbH, Singen, Germany) for 30 min at room temperature. On the time scale applied for the dielectric experiments, phase separation effects between LC and DNPs were not observed on a macroscopic scale.

### 3.4. Dielectric Measurements

The real and imaginary components (ε’ and ε”) of the complex dielectric function of NP, P, and DNP doped LC mixtures were measured. In this report, relative permittivities will be considered. The measurements were performed at room temperature (20 °C) in a frequency range from 0.1 Hz to 1 MHz with an impedance analyzer ModuLab-MTS test system from Solartron Analytical, Ametek, Berwyn, PA, USA. For preliminary tests, two amplitudes of the oscillating voltage were tested: 0.1 and 1 V.

**Figure 7:**
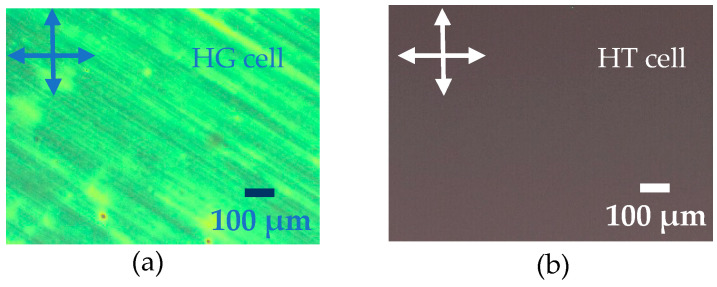
Textures of a purified LC mixture in (**a**) homogeneous and (**b**) homeotropic alignments. The micrographs are recorded by POM under cross-polarized condition at room temperature.

The measurements were performed using commercial (standard) cells of 20 µm thickness manufactured by AWAT, Warsaw, Poland. These cells are composed of two glass plates, each one is covered on its inner surface by a conductive coating (indium tin oxide: ITO), serving as electrode with a surface area of 0.25 cm^2^ and a sheet resistance of ~20 Ω/□. ITO electrodes are widely used for dielectric measurements and LCs devices. They are transparent to visible light and chemically stable [46,47]. The cells are also coated with polyimide to obtain homogeneous and homeotropic alignments. NP, P, and doped LC mixtures were inserted into the cells by capillary action at a temperature of 90 °C, where the LCs present an isotropic phase. Figure 7 illustrates the textures of a P LC mixture in homogeneous and homeotropic alignments, using the same cells employed for dielectric measurements.

The uniform and bright color throughout the texture exhibited in Figure 7a confirms the homogeneous alignment of the LCs in the cell. In contrast, Figure 7b reveals a totally dark micrograph corroborating the homeotropic alignment of LCs.

## 4. Conclusions

The dielectric properties of non-purified, purified, and doped recycled LC mixtures with different DNP concentrations (0.05, 0.1, and 0.2 wt%), in homogeneous and homeotropic alignments, have been investigated at room temperature using an impedance analyzer. Non-purified LC mixtures show positive values of dielectric anisotropy in the frequency range comprised between 20 and 10^5^ Hz. It has been demonstrated that a purification process is mandatory to improve the dielectric properties of these recycled LC mixtures. The addition of DNPs to these LC mixtures allowed to decrease the ionic conductivity due to the trapping of ionic impurities on their surface. A small amount of DNPs (0.05 wt%) has been shown to reduce the ionic conductivity of the sample by two orders of magnitude, regardless of the anchoring conditions, compared to the non-purified samples. Consequently, such recycled LC material could be upgraded for other uses (LC screens, smart windows).

## Figures and Tables

**Table 1:** Fitting parameters obtained from experimental data of σ’using Jonscher’s law.

Sample	σ_DC_ (S·m^−1^)		n		fc (Hz)		R^2^	
	HT	HG	HT	HG	HT	HG	HT	HG
NP	5.8 × 10^−8^ ± 3.5 × 10^−11^	2.9 × 10^−8^ ± 1.6 × 10^−11^	1.799 ± 0.010	1.728 ± 0.01	11,642.5 ± 133.50	26,951.9 ± 213.23	0.9996	0.9998
P	5.1 × 10^−8^ ± 2.7 × 10^−12^	2.5 × 10^−9^ ± 1.3 × 10^−12^	1.760 ± 0.011	1.750 ± 0.01	1798.4 ± 38.45	5719.6 ± 71.57	0.9993	0.9997
P + 0.05D	1.3 × 10^−10^ ± 1.1 × 10^−13^	3.0 × 10^−11^ ± 2.5 × 10^−14^	1.605 ± 0.030	0.7890 ± 0.02	352.2 ± 16.66	56.1 ± 5.77	0.9961	0.9930
P + 0.1D	1.1 × 10^−10^ ± 9.6 × 10^−14^	2.0 × 10^−11^ ± 2.4 × 10^−14^	1.425 ± 0.034	0.9078 ± 0.01	245.4 ± 14.64	43.4 ± 4.03	0.9944	0.9961
P + 0.2D	1.01 × 10^−10^ ± 1.3 × 10^−13^	1.7 × 10^−11^ ± 1.4 × 10^−14^	1.515 ± 0.035	0.7822 ± 0.01	214.2 ± 14.27	21.1 ± 1.52	0.9938	0.9983

## Data Availability

Data set presented in this study is available in this article.

## Supplementary Materials

Figure S1: Relative permittivity of three purified LC mixtures as a function of frequency. Measurements were taken at 1 V and room temperature (20 °C) using 20 µm cells in homogeneous and homeotropic alignments. P stands for purified LC mixtures.

## Sample Availability

Samples of the compounds are currently under use for other applications.
