# Supplementary material for: Dielectric Spectroscopy Analysis of Liquid Crystals Recovered from End-of-Life Liquid Crystal Displays
Source: Molecules. 2021 May 12;26(10):2873. doi: 10.3390/molecules26102873 (PMC8150612; doi:10.3390/molecules26102873)
Supplement: Supplementary file 1 [file molecules-26-02873-s001.zip › molecules-1204989-supplementary.pdf]

molecules

MDPI

Supplementary Materials

Dielectric Spectroscopy Analysis of Liquid Crystals Recovered from End-of-Life Liquid Crystal Displays

Ana Barrera [1], Corinne Binet [1], Frédéric Dubois [2], Pierre-Alexandre Hébert [3], Philippe Supiot [1], Corinne Foissac [1] and Ulrich Maschke [1,*]

[1] Univ. Lille, CNRS, INRAE, Centrale Lille, UMR 8207—UMET—Unité Matériaux et Transformations, F-59000 Lille, France; ana-luisa.barrera-almeida@univ-lille.fr (A.B.); corinne.binet@univ-lille.fr (C.B.); philippe.supiot@univ-lille.fr (P.S.); corinne.foissac@univ-lille.fr (C.F.)
[2] Univ. Littoral Côte d'Opale, UR 4476, UDSMM, Unité de Dynamique et Structure des Matériaux Moléculaires, France; frederic.dubois@univ-littoral.fr
[3] Univ. Littoral Côte d'Opale, UR 4491, LISIC, Laboratoire d'Informatique Signal et Image de la Côte d'Opale, France; pierre-alexandre.hebert@univ-littoral.fr
* Correspondence: ulrich.maschke@univ-lille.fr; Tel.: +33 3 20 33 63 81

Supplementary Material:

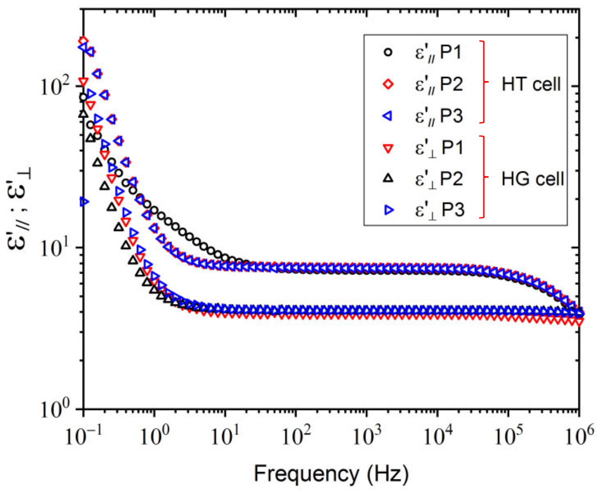

Figure S1. Relative permittivity of three purified LC mixtures as a function of frequency. Measurements were taken at 1 V and room temperature (20 °C) using 20 μm cells in homogeneous and homeotropic alignments. P stands for purified LC mixtures.
